# Mutagenesis in Rice: The Basis for Breeding a New Super Plant

**DOI:** 10.3389/fpls.2019.01326

**Published:** 2019-11-08

**Authors:** Vívian Ebeling Viana, Camila Pegoraro, Carlos Busanello, Antonio Costa de Oliveira

**Affiliations:** Centro de Genômica e Fitomelhoramento, Faculdade de Agronomia Eliseu Maciel, Departamento de Fitotecnia, Universidade Federal de Pelotas, Campus Capão do Leão, Rio Grande do Sul, Brazil

**Keywords:** genetic variability, mutagenesis, functional genomics, random mutations, targeted mutations, mutation detection, DNA repair, *Oryza sativa* L

## Abstract

The high selection pressure applied in rice breeding since its domestication thousands of years ago has caused a narrowing in its genetic variability. Obtaining new rice cultivars therefore becomes a major challenge for breeders and developing strategies to increase the genetic variability has demanded the attention of several research groups. Understanding mutations and their applications have paved the way for advances in the elucidation of a genetic, physiological, and biochemical basis of rice traits. Creating variability through mutations has therefore grown to be among the most important tools to improve rice. The small genome size of rice has enabled a faster release of higher quality sequence drafts as compared to other crops. The move from structural to functional genomics is possible due to an array of mutant databases, highlighting mutagenesis as an important player in this progress. Furthermore, due to the synteny among the Poaceae, other grasses can also benefit from these findings. Successful gene modifications have been obtained by random and targeted mutations. Furthermore, following mutation induction pathways, techniques have been applied to identify mutations and the molecular control of DNA damage repair mechanisms in the rice genome. This review highlights findings in generating rice genome resources showing strategies applied for variability increasing, detection and genetic mechanisms of DNA damage repair.

## Introduction

Rice (*Oryza sativa* L.), among all crops, has displayed the highest advances in functional genomics in recent decades. It is a diploid species, with a small genome in comparison to other cultivated cereals (Moin et al., 2017). In addition, the wide use of genetic transformation techniques, the synteny with other crop species, and a diversified source of related and closely related germplasm contributes enormously to the advantages in using rice as a genetic system for functional analyses (Lo et al., 2016). However, elite rice cultivars exhibit narrow genetic variability due to the repeated use of similar genotypes, i.e., with the same ideotype in crossing blocks. Understanding the genetic basis and the function of a given gene can help breeders to develop new, more productive and stress tolerant cultivars. One has to keep in mind that most of the agronomically important traits are of complex inheritance and therefore more difficult to improve. In this case, the mutant or variant allele can be detected and easily introgressed by performing Genome Wide Association Studies (GWAS) in populations including mutant genotypes.

Mutations can be used as a tool for gene functional studies and to create genetic variability. The analysis of mutants by forward genetics, i.e., from phenotype to gene, or by reverse genetics, i.e., from gene to phenotype, can be used to understand gene function (Lo et al., 2016). However, spontaneous mutation rates in higher plants are low, ranging from 10^−5^ to 10^−8^ (Jiang and Ramachandran, 2010). Thus, mutagenesis is an important strategy to increase mutation frequency (Maluszynski et al., 2000; Da Luz et al., 2016) enabling studies of functional genomics and the development of new genotypes.

Mutations can be randomly induced by genotoxic agents or by DNA insertions, and both can lead to gene gain or loss of function. Another form is by inducing direct mutations, in order to change the structure of a target gene. In rice, some important mutant collections created with the aim of functional studies, have been reported and a mutant library list was provided by Hirochika et al. (2004). Within the library, one can find mutant collections for randomly induced mutations (Maluszynski et al., 2000) and insertional mutations by transposons (van Enckevort et al., 2005), retrotransposon (reviewed in Hirochika, 2010), T-DNA insertions (Zhang et al., 2006; Hsing et al., 2007; Piffanelli et al., 2007) and even Arabidopsis mutant lines expressing rice full-length cDNA (Sakurai et al., 2011). The mutants developed can help to define a pathway or be directly used in agriculture, a classic example being the imidazolinone herbicide-resistant rice cultivars (Sudianto et al., 2013).

Considering the importance of developing rice mutants towards the understanding of gene function and to create genetic variability, this review reports on the most recent advances on the identification of gene function and taps the genetic reservoir obtained through mutations in the rice genome.

## Random Mutations

**Figure 1:**
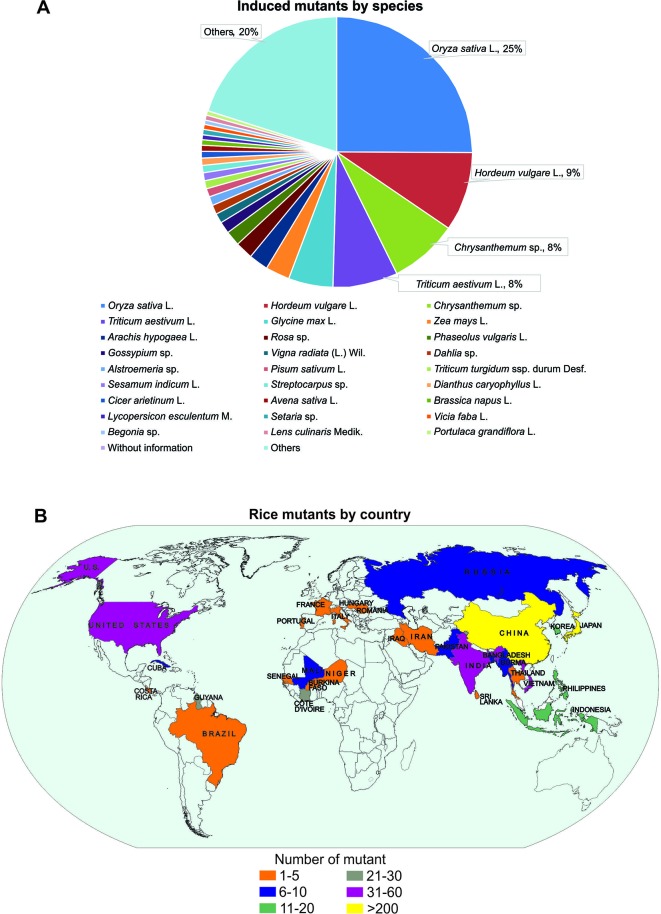
Mutant distribution according to Food and Agriculture Organization of the United Nations/International Atomic Energy Agency – Mutant Variety Database (FAO/IAEA-MVD, 2019). **(A)** Distribution of mutants by species; **(B)** Distribution of mutants by country (map).

Mutations are the major source of genetic variability and artificial mutations can be induced by mutagens (Wei et al., 2013; Oladosu et al., 2016). There are three major tools for mutagenesis: biological agents such as transposons, retrotransposons, and T-DNA; physical agents such as ionizing radiations or chemical agents such as alkylating agents; and azides (reviewed in Serrat et al., 2014). The Food and Agriculture Organization of the United Nations/International Atomic Energy Agency – Mutant Variety Database (FAO/IAEA-MVD) data (2019) reports on the developed and officially released mutants, a total of 3,275 accessions from 225 species. The released mutants are mainly composed of cereal species (47%) with the majority (25%) being rice mutants (**Figure 1A**). The 823 mutant rice events are distributed in 30 Countries, with China and Japan responsible for 35.6% and 26.8% of this total, respectively (**Figure 1B**).

In rice, changes in the gene structure can be randomly caused by rupturing the DNA through physical or chemical agents (Jain, 2010; Wei et al., 2013;Yang et al., 2013). The mutagenesis in rice is advantageous due to its small genome, i.e., a small population is required to saturate the whole genome and to provide a larger allelic series for use in mutagenesis (Wu et al., 2005; reviewed in Wang et al., 2013). Random mutations caused by physical and chemical agents have been applied to create genetic variability and for gene functional studies in rice (**Table 1**).

**Table 1:** Chemical and physical mutagens applied for induction of random mutations in rice.

	Method	Mechanism of mutation	Mutation frequency	Dose	Mutants produced	Pros	Cons	
	Chemical							
	EMS	Guanine alkylation, G/C to A/T transitions or G/C to C/G or G/C to T/A transversions.	2-10 mutations each Mb (Till et al., 2007)	0.2–2.0%	Plant development and metabolism (Feldman et al., 2014; Feldman et al., 2017)	Improve one or two traits with decreased rates of undesirable changes.	Manipulation of the mutagen	
					Herbicide resistance (LSU AgCenter)			
					Abiotic stress tolerance (Panigrahy et al., 2011; Poli et al., 2013; Mohapatra et al., 2014; Xu et al., 2017a)			
	MNU	Guanine and cytosine alkylation, G/C to T/A transitions.	1 mutation each 135 Kb (Satoh et al., 2010)	0.25 - 1.00 mM	Plant development and metabolism (Cha et al., 2002; Miyoshi et al., 2004; Ikeda et al., 2005; Nagasaki et al., 2007; Kurakawa et al., 2007; Qiao et al., 2010; Akter et al., 2014; Chen et al., 2015; Zhou et al., 2017; Inahashi et al., 2018)			
					Nutritional quality (Sakata et al., 2016; Kim et al., 2018b)			
					Plant chemical element transporters (Ma et al., 2007; Shao et al., 2017; Kim et al., 2018a)			
					Biotic stress resistance (Jung et al., 2005; Busungu et al., 2016)			
					Yield and quality improvement (Itoh et al., 2017; Long et al., 2017; Seo et al., 2017)			
	AS	Generates azidoalanine causing G/C to A/T transitions.	1.4 - 2.9 mutation each Mb (Tai et al., 2016)	1 - 10 mM	Industrial quality (Mo et al., 2013);			
					Nutritional improvement (Jeng et al., 2011; Jeng et al., 2012)			
					Abiotic stress tolerance (Hussain et al., 2012; Lin et al., 2016)			
					Yield and quality improvement (Lin et al., 2011; Lin et al., 2014))			
	Colchicine	Chromosome doubling, affects the microtubules promoting symmetric cell division.	−	0.04 - 0.3%	Nutritional quality (Tu et al., 2014)			
					Regulatory mechanism of genome duplication (Cai et al., 2007; Zhang et al., 2015)			
					Abiotic stress tolerance (Tu et al., 2014)			
					Yield and quality improvement (Cai et al., 2007; Zhang et al., 2017; Guo et al., 2017)			
	DEP	Guanine and adenine alkylation, deletions (1Kb) and point mutations.	−	0.004% - 0.006%	Abiotic stress tolerance(Nakhoda et al., 2012)			
	**Physical**							
	γ rays	Single nucleotide substitution, inversion and deletion.	7.5×10^−6^ to 9.8×10^−6^ (Li et al., 2016c)	50 - 350 Gy	Plant development and metabolism (Hirano et al., 2010; Han et al., 2012; Smillie et al., 2012; Li et al., 2017a; Mbaraka et al., 2017; Li et al., 2018c)	Higher DNA damage, affecting many traits.	Necessary specialized physical structure.	
					Industrial quality (Kong et al., 2015)			
					Nutritional quality (Chun et al., 2012; Hwang et al., 2014; (Sansenya et al., 2017)			
					Abiotic stress tolerance (Song et al., 2012; Joshi et al., 2016; Hwang et al., 2017)			
	IBR	Point mutation (deletion), inversion, translocation and insertion.	Survival rates from 70 to 90% mutation of 1.7%; 70% survival rates mutation of 2.0% (Yamaguchi et al., 2009)	Carbon 20 - 50 Gy (up to 220 MeV) Neon 10 Gy (60-80 keV/µm)	Nutritional quality (Ishikawa et al., 2012) Plant development and metabolism (Abe et al., 2002; Maekawa et al., 2003; Hayashi et al., 2007; Phanchaisri et al., 2007; Phanchaisri et al., 2012)			
FNI		A/T to G/C transition, insertion, inversion, duplication and deletion.	28-78 genome mutations (Li et al., 2016a)	20 Gy	Industrial quality (Mei, 2010)			
					Biotic stress resistance (Bart et al., 2010; Chern et al., 2014; Chern et al., 2016)			
					Abiotic stress tolerance (Ruengphayak et al., 2015)			
CRR		−	−	15 days space environment	Plant development and metabolism (Kim et al., 2012)			

EMS, Ethyl methane sulfonate; MNU, N-methyl-N-nitrosourea; SA, Sodium azide; DEP, Diepoxybutane; IBR, Ion beam radiation; FNI, Fast-neutron irradiation; CRR, Cosmic-ray radiation.

Indeed, the degree of mutation depends on the tissue, dosage and time of exposure (Parry et al., 2009). An important step when obtaining a mutant population, either by chemical or by physical agents, is the LD_50 _determination (reviewed in Talebi et al., 2012). The highest mutation frequency is expected to occur in the treatment that kills 50% of the treated material (Jain, 2010). In this sense, the LD_50_ corresponds to the dose that kill half of the treated population (reviewed in Talebi et al., 2012). In rice, mutants were developed from immature embryos (Ookawa et al., 2014), calli obtained from seeds (Serrat et al., 2014) and cell suspension cultures (Chen et al., 2013). Seeds are, however, easier to handle and do not require a specialized structure, and is therefore, the most widely used material (Da Luz et al., 2016;Oladosu et al., 2016). When seeds are mutagenized, it is important to standardize the seed mutagenic absorption through the pre-soaking of the seed in distilled water, since it also activates the seed metabolism and helps the mutagen action.

**Figure 2:**
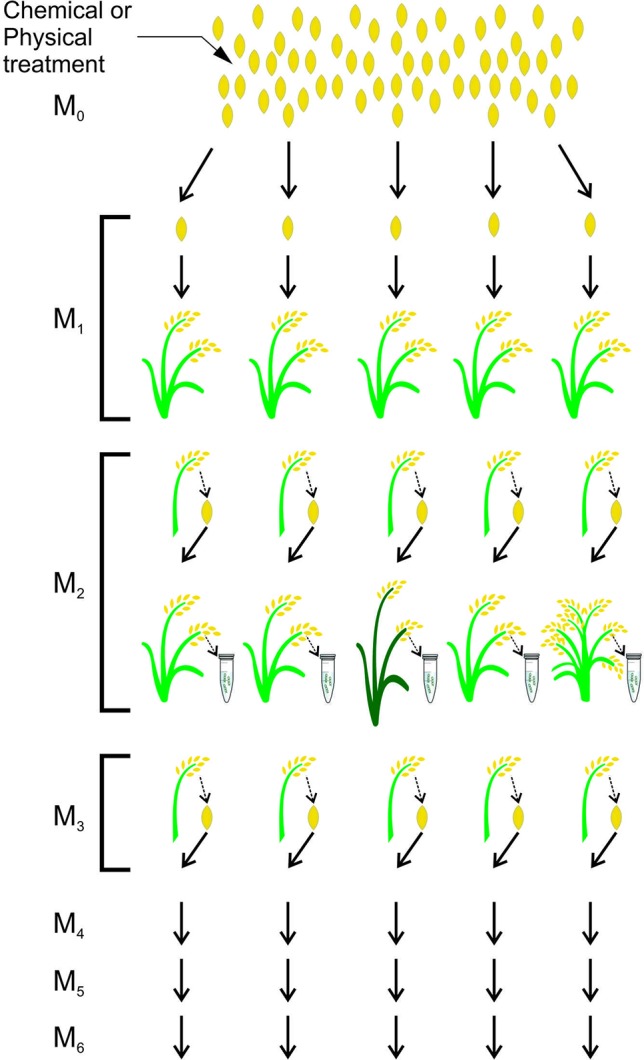
Scheme for obtaining a mutant population in rice. Mutagenesis is performed with a chemical or physical seed treatment given to an M_0_ population. The M_1_ plants originate from each treated M_0_ seed. Seeds from M_1_ plants will form the M_2_ population in which DNA analyses will be performed to find mutations. Seeds from M_2_ plants will form the M_3_ population and the next populations will be obtained in the same way. adapted from (Tai, 2017)

In plant breeding, the induction and identification of seed mutations is a simple process. Once the mutant population is obtained and the mutation is identified, seeds from the mutant plant compose the next generation in which the phenotype will be analyzed in order to search for mutation effects (Henry et al., 2014) (**Figure 2**).

### Random Mutations Induced With Chemical Agents

The advantage of using chemical agents is the possibility to improve one or two traits while avoiding undesirable changes (Jeng et al., 2011). The chemical mutagens ethyl methanesulfonate (EMS), methyl methanesulfonate (MMS), hydrogen fluoride (HF), sodium azide (SA), N-methyl-N-nitrosourea (MNU), and hydroxylamine (H_3_NO) are the chemicals most frequently used in plants (Parry et al., 2009). The effects of chemical treatment are silent or missense mutations (50%) while only 5% of nonsense mutations are observed (Nawaz and Shu, 2014).

#### Ethyl Methanesulfonate

Ethyl methanesulfonate (EMS) causes mutations through the alkylation of guanine bases leading to (mis)matches with thymine rather than cytosine, resulting in transitions of G/C to A/T (reviewed in Talebi et al., 2012). However, with less frequency, EMS can cause G/C to C/G or G/C to T/A transversions through 7-ethylguanine hydrolysis or A/T to G/C transitions through mismatches of 3-ethyladenine (reviewed in Serrat et al., 2014). In rice, EMS mutagenesis involves seed soaking in a solution with a known EMS concentration, usually 0.2 to 2.0%. The time in the soaking solution ranges from 10 to 20 h and considers the sensibility or the lethal curve of the genotype used (reviewed in Talebi et al., 2012). Luz and co-workers (2016) generated 340 rice mutant families applying EMS 1.5% for 2 h and a significant difference between the families was reported, indicating the efficiency of the dose and the time of exposition in generating mutants. EMS treatment was also reported to induce genetic variability in Basmati rice (Wattoo et al., 2012). For EMS treatment, presoaked seeds were immersed in EMS 0.5, 1.0, 1.5 and 2.0% for 6 h, suggesting that the 0.5% to 1.0% doses for 6 h, can induce mutations in rice. Since EMS mutagenesis causes a higher number of non-lethal point mutations, a relatively small population, ca. 10,000 plants, is necessary to saturate the genome with mutations. In diploid organisms, EMS treatment (1.5%) can induce 1 mutation every 294 Kb (Till et al., 2007).

Reports have demonstrated that the application of EMS improves agronomically important traits. One of the focuses in rice mutagenesis is to obtain a genotype with C4 photosynthesis. The mutagenesis with EMS allowed one to obtain mutants affecting important traits such as increased vein number and the photosynthetic rate in leaves and reduced mesophyll interveinal cells (Feldman et al., 2014; Feldman et al., 2017). Traits involved in abiotic stress tolerance were also obtained. Among these, traits for drought tolerance such as root length, the volume of roots, and root weight were increased (Mohapatra et al., 2014). Heat and drought tolerance were associated to low ROS accumulation, which is related to these stresses, and mutants showing higher chlorophyll retention were also heat tolerant (Panigrahy et al., 2011). Furthermore, a heat tolerant mutant with higher photosystem II efficiency was identified (Poli et al., 2013). These studies demonstrated the potential use of the mutation for higher yielding, stress resilient genotype development. On the other hand, EMS mutagenesis was used in reverse genetics studies to identify gene function. The *OsHAC4* gene that codes for rhodanase-like protein was identified and reported to be involved in arsenate tolerance mechanism and in arsenic accumulation in rice (Xu et al., 2017a).

#### N-Methyl-N-Nitrosourea

**Figure 3:**
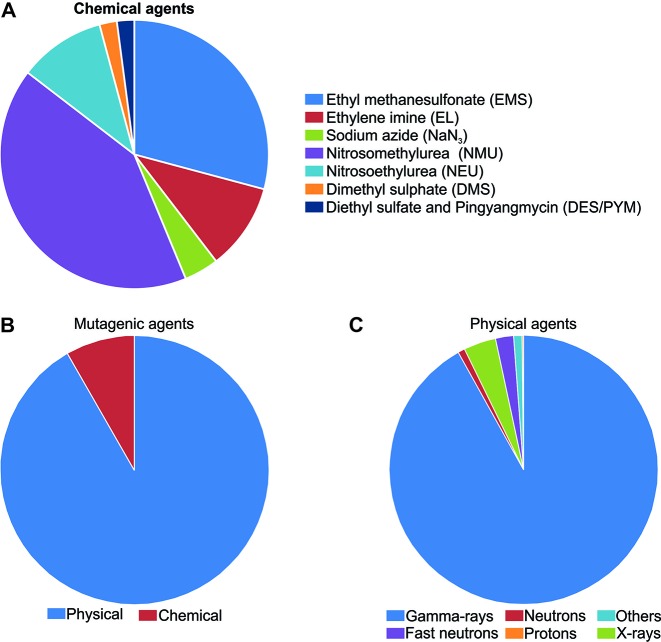
Mutagens applied in rice mutagenesis according to Food and Agriculture Organization of the United Nations/International Atomic Energy Agency – Mutant Variety Database (FAO/IAEA-MVD, 2019). **(A)** Chemical mutagens; **(B)** Chemical and physical agents; **(C)** Physical mutagens.

Undoubtedly, N-methyl-N-nitrosourea (MNU) is the most applied mutagen in rice (FAO/IAEA, 2019) (**Figure 3A**). MNU is a monofunctional alkylating agent and presents high reactivity with oxygen atoms in the DNA molecule. Mutation initiates with the addition of alkyl groups to nitrogenous bases, mainly guanine and cytosine. The generation of *O* *^6^*-alkylguanine, induced by MNU treatment, promotes G/C to A/T transitions through mismatches with thymine during DNA replication (reviewed in Satoh et al., 2010). However, *O* *^6^*-alkylguanine occurrence is not random and is influenced by the guanine 5’ and 3’ flanking sequence, DNA conformation and chromatin structure. If the 5’guanine is flanked by a 5’adenine and 3’cytosine, it shows a 10-fold increase in chance to form *O* *^6^*-alkylguanine (Sendowski and Rajewsky, 1991). It was reported that mutagenesis with MNU in rice is more efficient in developing cells than in dry seeds (Satoh et al., 2010). When treatment with MNU is applied, one mutation every 135 Kb is expected (Satoh et al., 2010).

Mutagenesis with MNU treatment has been widely applied and affects a range of plant physiological processes leading to the discovery of gene functions and the increase of genetic variability. With molecular and phenotypic markers, the locus *stg*(t) (responsible for the Stay Green trait) was mapped on chromosome 9 (Cha et al., 2002). *Spotted Leaf Mutants* (*spl28*) were identified and displayed the formation of hypersensitive lesions leading to the initiation of leaf senescence (Qiao et al., 2010). The gene *Young Seedling Stripe2* (*YSS2*) was also identified and characterized to be involved in chloroplast biosynthesis (Zhou et al., 2017). Additionally, the *Plastochron1* (*Pla1*) mutant, which is involved in leaf primordia development and affects the timing of leaf vegetative growth, was also identified (Miyoshi et al., 2004). The *LGF1* (*Leaf Gas Film 1*) gene was found to control leaf hydrophobicity and the formation of gas films (Kurokawa et al., 2018).

The silicon efflux transporter gene *Low Silicon Rice 2* (*Lsi2*) was also identified through MNU mutagenesis (Ma et al., 2007). Also related to transport, the *Zebra3-1* (*z3-1*) gene was characterized to be involved on citrate transport and distribution during leaf development (Kim et al., 2018a). In addition, mutant lines with higher cadmium accumulation showed low *OsHMA3* expression that suggested the involvement of this gene in cadmium accumulation (Shao et al., 2017). Genes involved in auxin efflux and signaling were identified. The *Ospin-Formed2* (*Ospin2*) gene, that codes for auxin-efflux carrier proteins (Inahashi et al., 2018) and *Giant Embryo* (*GE*) that controls the expression of auxin and cyclin responsive genes (Chen et al., 2015), were identified.

Findings using MNU also help to understand rice responses against biotic stresses. The *Blast Lesion Mimic* (*BLM*) was identified to confer resistance against fungus (Jung et al., 2005). A different mutant line, XM14, showed resistance to six races of *Xanthomonas oryzae* pv. *oryzae*, in which the *Xa42* gene*, *responsible for resistance responses, was identified (Busungu et al., 2016).

In addition, through MNU mutagenesis, a better understanding of the embryogenic processes in rice was achieved. The *Shootless2* (*SHL2*), *SHL4/Shoot Organization2* (*SHO2*), and *SHO1* genes were reported to be involved in normal shoot apical meristem (SAM) development (Nagasaki et al., 2007). The *Lonely Guy* (*LOG*) was also identified, activating cytokinin and regulating SAM activity (Kurakawa et al., 2007).

Mutation induction with MNU also focused on grain yield and quality traits, which undoubtedly are the most important agronomic traits. The *Aberrant Panicle Organization1-1* (*APO1-1*) allele was identified involved in inflorescence architecture, floral organ identity and leaf production (Ikeda et al., 2005). Also, the *Panicle Apical Abortion* (*PAA-Hwa*) gene was reported to be involved in panicle identity (Akter et al., 2014). Through the introduction of the *indica* *SSIIa* and *GBSSI* genes in a *BEIIb* *japonica* mutant genotype, it was possible to generate lines displaying higher resistant starch and amylose contents (Itoh et al., 2017). The *Floury Endosperm8* (*FLO8*) gene was identified as a regulator of starch biosynthesis related genes (Long et al., 2017). Regarding seed yield and shape, the *Dense and Erect Panicle 2-3* (*DEP2-3*) was reported to play a role in rice seed size and shape (Seo et al., 2017). The rice grain oil content was modified by MNU treatment and mutant lines showing increased triacylglycerol content were also identified (Sakata et al., 2016). The content of flavonoids, phenolic compounds and antioxidant activity was also increased in rice seeds (Kim et al., 2018b). The studies described clearly illustrate the efficiency in the induction of mutation in rice treated with MNU and its potential application in rice functional genomics and improvement.

#### Sodium Azide

Sodium azide (SA- NaN_3_) is an ionic compound and its mutagenicity is mediated through an organic metabolite (analogous to L-azidoalanine) of the azide compound generated by O-acetylserine sulfhydrylase enzyme (Gruszka et al., 2012). This metabolite enters the cell nucleus and interacts with the DNA, creating point mutations in the genome. In rice, SA predominantly causes transitions from G/C to A/T and similar sequences at the mutation site were identified as 5’-GGR-3 ‘ (Tai et al., 2016). The mutation rate identified was 1.4 to 2.9 mutations per Mb. It is known that the mutagenic effect of SA depends strongly on the solution pH and can be potentiated when germinated seeds are treated (Gruszka et al., 2012).

Important findings were obtained through SA treatment towards rice improvement. The Suweon 542 is a mutant rice with potential application on rice flour production, showing floury grains and small and less degraded starch particles (Mo et al., 2013). In this mutant, the recessive locus *flo7(t)* explains 92.2% of the grain flour variation. SA treatment in red rice also increased the amounts of proto-cyanidins, γ-oryzanol, vitamin E and iron accumulation (Jeng et al., 2011). Increased iron and zinc amounts in polished grains, starch and amylose contents and production of new aromatic rice were also observed (Lin et al., 2011, Jeng et al., 2012, Lin et al., 2014). Abiotic stress tolerant lines were also obtained with SA treatment. The *salt hypersensitive 1* (*shs1*) mutant was reported to play a role in antioxidant metabolism and Na^+^ homeostasis under salt stress (Lin et al., 2016). The *tms8* gene was found to be involved in genetic male sterility induced by temperature (Hussain et al., 2012).

Although less prominent than EMS and MNU, SA is an alternative tool for mutagenesis and can create variability for rice functional genomics and breeding, respectively.

#### Colchicine for Polyploid Induction

Polyploidization is an important event for plant evolution. When the ploidy is increased, the plant yield tends to increase, and sometimes organs tend to get bigger. This phenomenon suggests polyploidization as an improvement strategy towards the production of a better rice plant. Colchicine is a chemical mutagen generally used to induce chromosome doubling. It is a mitogen disrupting agent which affects the microtubules that organize chromosomes in one side of the cell promoting symmetric cell division (reviewed in Alemanno and Guiderdoni, 1994). The induction of polyploidization in rice is generally performed through colchicine treatment in callus derived from anthers or seed cultures (Alemanno and Guiderdoni, 1994; Premvaranon et al., 2011; Zhang et al., 2017). In some cases, polyploidy induction causes genome rearrangements which can negatively affect seed production. After a long period of investigation, two polyploid rice lines with *Polyploid Meiosis Stability* (*PMeS*) genes were obtained from progenies of polyploid hybrids from *indica-japonica* crosses. The PMeS-I and PMeS-II lines showed different traits compared to other polyploid lines such as increased seed yield and meiosis stability (Cai et al., 2007).

With the aim of exploring heterosis in rice, two rice polyploid lines with photoperiod- and thermo-sensitive genic male sterility were developed. These lines showed high cross pollination ability, changes in fertility, and high combination ability. Hybrids obtained from these lines showed high heterosis and the potential to increase rice grain yield and quality (Zhang et al., 2017). The transcriptome of rice neo-tetraploids revealed a group of genes associated with fertility and heterosis. Among them, transcription factors, methyltransferases, photosynthesis related genes, metabolic processes, transport, fertility, resistance, epigenetic elements, meiosis and retrotransposons were identified showing the complexity of regulatory mechanisms associated with fertility and heterosis (Guo et al., 2017). This demonstrates that polyploidy induction may be a way to potentialize heterosis in self crossing plants such as rice.

To understand the regulatory mechanisms related to genome duplications in rice, different studies were performed. DNA methylation analyses in a rice autotetraploid revealed class II transposable element (TE) hypermethylation at CHG and CHH sites. The increase in TE methylation inhibits its transposition, stabilizing the chromosomal integrity, and can suppress the expression of neighboring genes, potentially decreasing the deleterious effects of the dosage of these genes (Zhang et al., 2015). The authors suggested that the hypermethylation can be a response factor to “genome shock” to help neo-autopolyploids in adapting to effects caused by the dosage of these genes.

Regarding abiotic stress responses, chromosomal duplication in rice resulted in increased tolerance to salinity (Tu et al., 2014). Also, autotetraploids presented more salinity stress tolerance during germination and reduced mortality rate during the initial growth. In addition, autotetraploid rice presented higher proline and soluble sugar contents, as well as decreasing malondialdehyde content when compared to diploid rice (Jiang et al., 2013).

A synthetic allopolyploid rice (DSAR) technology was developed to produce an allopolyploid rice which involves hybridization with distant genotypes following chromosome doubling (Zhang et al., 2014). DSAR technology comprises wild rice crossing followed by embryo rescue and chromosome doubling with colchicine treatment.

### Random Mutations Caused by Physical Agents

Physical agents are the most used mutagens in rice (FAO/IAEA, 2018), corresponding to 91.6% of the reports found (**Figure 3B**). Ionizing radiation can be divided in two classes according to the linear energy transfer. The higher rates of linear energy transfer occur in alpha, neutrons and heavy ion beams while small rates are found in γ-rays, X-rays and electron beams (Hwang et al., 2014). Ionizing radiation produces reactive oxygen species (ROS) which interact with the DNA causing oxidative damage, nucleotide changes and single strand or double strand breaks (reviewed in Roldán-Arjona and Ariza, 2009).

#### Gamma-Rays

Gamma-rays (γ-rays) have been widely used to generate mutants in rice, ca. 92% of the rice mutants obtained with physical agents, were generated with γ-rays (FAO/IAEA, 2018) (**Figure 3C**). Small deletions (1–16 pb) were the most frequent effect of γ-rays on the rice genome, however larger deletions (9.4–129.7 kb) and large fragment inversions (1284.8 – 3208.5 kb) were also detected (Morita et al., 2009). Indels and single base substitution were also identified, with a higher frequency of heterozygous when compared to homozygous mutations (Li et al., 2016c). Also, the treatment of dry seeds resulted in heritable mutations at frequencies of 7.5×10^−6^ to 9.8×10^−6^ (Li et al., 2016c). Reports have indicated that γ-rays generate genetic variability in a wide range of rice genotypes, being an effective tool for rice improvement (Kole et al., 2008; Harding, 2012).

Mutants induced with γ-rays help with the identification of genes related to specific physiological processes in rice plants. The *necrotic lethality1* (*nec1*) gene, when heterozygous, controls leaf necrosis processes (Mbaraka et al., 2017). Analyses in the *early-senescence-leaf* (*esl*) mutant showed the involvement of the NADPH oxidase in the abscisic acid (ABA) signaling through O_2_^-^ radical production (Li et al., 2018a). Gamma-rays were able to create genetic variability for abiotic stress tolerance, such as salinity in ST-87 and ST-301 lines (Song et al., 2012), as well as plant height, tiller number, shoot and root weight, total biomass and panicle length (Joshi et al., 2016). The *arsenic-tolerant type 1* (*aatt1*) showed tolerance to arsenic in the soil (Hwang et al., 2017).

Changes in rice grain content were also reported. The *OASA1* mutant showed increased tryptophan storage, which enabled researchers to identify that rice tryptophan accumulation is influenced by the *OASA1* gene structure (Chun et al., 2012). The MRXII mutant displayed elevated tocopherol content which was related to SNPs created in the *OsVTE2* promoter, which generate a MYB transcription factor binding site (Hwang et al., 2014). An aromatic rice was also obtained by γ-rays. These lines showed increased 2-acetyl-1-pyrroline (2AP) and decreased γ-aminobutyric acid (GABA) content (Sansenya et al., 2017). GM077 and GM645 mutants showed lower and higher amylose content, respectively, indicating that γ-rays also influenced starch storage in rice grains (Kong et al., 2015).

DNA changes using γ-rays were also the target of a project aiming to turn rice into a C4 plant. The *altered leaf morphology* (*alm*) showed reduced leaf interveinal distances, which is determined by the mesophyll cell size, an important anatomical adaptation to obtain a Kranz anatomy, typical in C4 plants (Smillie et al., 2012). Also, regarding cell structure, *Brittle Culm 3* (*BC3*) was characterized to be involved in secondary cell wall biosynthesis (Hirano et al., 2010). The gene *OsCLD1/SRL1* (*Semi‐Rolled Leaf 1*) was characterized in the cell wall synthesis, epidermal integrity and water homeostasis pathways (Li et al., 2017a). *Zebra2* (*z2-2*) was identified associated with the tetra-*cis*-lycopene accumulation (Han et al., 2012).

#### Ion Beam Radiation

Ion beam radiation (IBR) differs from γ-rays concerning the linear energy transfer, and produces important deletions, greater than 1Kbp (reviewed in Nawaz and Shu, 2014). IBR effects were verified in *Arabidopsis thaliana*. It causes point mutations (most of them deletions) and intergenic rearrangements as deletions, inversions, translocations and insertions (reviewed in Tanaka et al., 2010). The DL_50_ of Carbon ions was estimated in 30 Gy (220 MeV) for the genotype Nipponbare (Maekawa et al., 2003). After, doses up to 20 Gy (23-40 keV/μm) and 10 Gy (60-80 keV/μm) in Carbon and Neon accelerated ion radiation, respectively, were recommended for the genotype Nipponbare to produce plants with high survival rates, fertility and with high frequency of chlorophyll deficiency mutants (Maekawa et al., 2003; Hayashi et al., 2007). IBR induces mutations with high frequency at a relatively low dose and also induces a broad spectrum of phenotypes without affecting other plant traits being these an advantage in applying IBR for rice mutation (reviewed in Ishikawa et al., 2012).

Applying IBR, the role of some important genes was unveiled in rice. Mutations were obtained in rice irradiated with Carbon ions, i.e., the *OsNramp5* gene was changed, leading to a decreased Cadmium content in grains (Ishikawa et al., 2012). Nitrogen bombardment also causes variations in some important traits, such as plant height, leaf, tegument and pericarp color (Phanchaisri et al., 2007). *PKOS1*, *HyKOS1*(dwarf) and *TKOS4* (tall) mutants were obtained through bombardment with the low energy Nitrogen ion. It enabled the identification of the genes *OsSPY* and *14-3-3*, which act as negative regulator of gibberellins and repressor of the *RSG* (*Repression of Shoot Growth*), respectively (Phanchaisri et al., 2012).

#### Fast-Neutron Irradiation

Fast-neutron irradiation affects the DNA structure, damaging nitrogenous bases promoting standard breaks. Also, single nucleotide substitution, insertions and duplications were reported as an effect of fast-neutron irradiation (reviewed in Li et al., 2016a). Recently, 1,504 rice mutants generated by fast-neutron radiation on the Kitaake genotype were sequenced (Li et al., 2017b). More than 91,000 mutations were identified affecting 58% of the rice genes, with an average of 61 mutations in each line. These changes included single nucleotide substitutions, deletions, insertions, inversions, translocations and tandem duplications.

Despite the important DNA damage induced by fast-neutron irradiation, few reports have applied this tool as a source of variation in rice. However, important traits for rice grain quality were obtained. Changes in amylose content were detected in some mutant lines, while the irradiation did not alter the amylopectin structure (Mei, 2010). Tolerance to iron toxicity was obtained, fast neutron irradiation induced changes in the *FRO1* gene, responsible to increase tolerance and higher iron accumulation in grains (Ruengphayak et al., 2015). Seeds from a rice genotype overexpressing *NH1* (*NPR1 homologue 1*) gene treated with fast neutron irradiation provided information regarding immune responses against bacterial infection by *Xanthomonas oryzae* pv. *oryzae* (*Xoo*) (Bart et al., 2010). The *NH1ox* line showed *Xoo* resistance and the loss of function mutation of *SNL1* gene (Supressor of *NH1-mediated Lesion*) caused suppression of the resistance conferred by *NH1*. Lately, the *NH1ox-54* line was irradiated with fast neutron and new genes related with *SNL1* activity were identified (Chern et al., 2014). RNAi lines obtained for *CRK6* and *CRK10* (*cysteine-rich receptor-like kinases*) genes displayed the *snl1* phenotype indicating that these genes are required for the immune responses mediated by *NH1*. Recently in the *NH1ox-54* irradiated line, a mutant for *suppressor of NH1- mediated immunity 1* (*snim1*) which suppresses the resistance conferred by *NH1* was identified (Chern et al., 2016).

Fast-neutron beams are highly important for functional genomics; however, it has not yet been applied in rice breeding. The characterized genes can be used towards the development of new varieties, since genes involved in agronomically important traits such as disease resistance, abiotic stress tolerance and grain quality have been altered.

### Random Mutations Induced by Other Chemical and Physical Agents

Cosmic-ray radiation has been applied to induce genetic variability in rice (Kim et al., 2012). Seeds irradiated with γ-, cosmic-rays or IBR have been obtained. The ROS accumulation, peroxidases, ascorbate peroxidases and dismutase superoxide activity were increased in the three treatments, however only γ- and cosmic rays were able to decrease the carotenoids and chlorophyll contents. In addition, cosmic-ray radiation was able to change the upregulated and downregulated gene profiles compared to other types of radiation.

Diepoxybutane (DEB), a bifunctional alkylating agent, acts on nitrogen 7 of the guanines and nitrogen 3, 6, 7 and 9 of the adenines, promoting DNA-DNA and DNA-protein linkages (reviewed in Nakhoda et al., 2012). It is predicted that DEB cause small deletions (1Kb) and point mutation (reviewed in Wu et al., 2005). Mutants developed with DEB treatment, showed variability for salinity tolerance (Nakhoda et al., 2012). Tolerant mutants absorbed less Na^+^ and more K^+^, increased shoot biomass and elevated survival rate compared to the wild-type.

Mutation is an important tool in creating genetic variability. In this sense, there are several agents being tested that provide different effects on DNA. These studies are extremely important since they demonstrate the range of possibility by which the structure of DNA can be modified.

### Random Mutations Induced by Combinations of Mutagens

Besides the chemical and physical mutagen’s application described above, studies have demonstrated the combined effect of these agents in increasing the frequency of mutations. The combination of chemical and physical agents was reported in rice treated with γ-rays followed by EMS seed soaking (Theerawitaya et al., 2011). Salinity tolerant mutants were isolated and were able to survive up to 15 days in 342 mM NaCl solution while sensitive plants were already affected by 171 mM NaCl after 5 days.

The combination of γ-rays, EMS and SA was also used in Basmati rice mutagenesis (Siddiqui and Singh, 2010). The combination of different doses promoted decreases in plant height, panicle size, panicle number and in the weight of 100 seeds. Rice seeds irradiated with γ-rays, electron beam or recurrent treatment with γ-rays, showed that electron beam radiation presented higher mutation frequency for chlorophyll content compared with γ-rays and recurrent treatment (Manikandan and Vanniarajan, 2017).

## Insertional Mutagenesis

Insertional mutagenesis is based in T-DNA (transfer DNA), transposon and retrotransposon, which are randomly inserted into the genome generating a wide range of mutations (Sallaud et al., 2004). This strategy has been applied to produce mutant populations in rice. The identification of rice insertions is facilitated by the complete genome sequence and by the efficiency of *Agrobacterium tumefaciens* infection methods, which promotes the T-DNA insertion in the rice genome (reviewed in Wang et al., 2013). However, there is a concern with the somaclonal variation observed during *in vitro* conditions, a condition that could mask the induced mutations. In this sense, the *in vitro* variation frequency in transgenic rice was shown to be low and non-significant when calli are transformed using *A. tumefaciens* (Wei et al., 2016).

### T-DNA Insertion Mutagenesis

Undoubtedly, one of the most widely used tools for gene function identification is through T-DNA insertions, which can cause loss of function and through the observed phenotype, a direct response regarding their biological function (Lo et al., 2016). Otherwise, it can result in gain of function, as reported in the insertional mutant *OsHKT1;4*. This mutant presents a T-DNA insertion in the upstream region of the first ATG codon, generating elements in tandem repeat with nuclei sequence of the 35S promoter (Oda et al., 2018). This mutant, as well as other insertional mutants applied to rice functional genomics, can be obtained in one of the most important sources of T-DNA insertional mutants, the Rice Functional Genomic Express Database (http://signal.salk.edu/cgi-bin/RiceGE).

Meantime, an intriguing finding about the T-DNA insertions was reported in *Arabidopsis*. The *A. tumefaciens* transformation using floral dipping method (to avoid *in vitro* processes) was compared to the wild-type genotype. The genome was resequenced using Next Generation Sequencing (NGS) and rearrangements of the T-DNA insertions with small mutations not correlated with the final position of the insert were identified (Schouten et al., 2017). In addition, small as well as large deletions were reported specifically in the T-DNA insertion site, and a T-DNA piece without the edge sequence (T-DNA splinter) was identified. Although a phenotype is expected, this does not always occur for two reasons: firstly, because it is unknown where the T-DNA will be inserted and secondly because other modifications may occur (Schouten et al., 2017).

Recently, a robust review regarding T-DNA insertions in the rice genome was published (Lo et al., 2016). Thus, we will point out the most recent findings that complete the state of the art of this mutagenesis tool in rice. With the advance of biotechnology techniques, especially in the understanding of rice *in vitro* culture, thousands of insertional mutants have been developed. Through the T-DNA vectors carrying the reporter GUS (ခβ-glucuronidase) promoter-less, the development of 100,000 rice insertional mutants were reported. Among them, mutant lines with insertions in transcription factor coding genes (Wei et al., 2017). These mutants were applied to identify the preferred location regarding transcription factor action and can be used for functional analyses of these candidate genes. In the last two years, several insertional lines providing the functional identification of rice genes involved in many important traits have been described (**Table 2**).

**Table 2:** Biological mutagens applied for induction of random mutations in rice.

Method	Mechanism of mutation	Mutation frequency	Mutants produced	Pros	Cons
T-DNA*	Random sequence insertion	88.8% (6645 insertions of 7480) (Sallaud et al., 2004)	Plant development and metabolism (Basu et al., 2017; Chen et al., 2017a; Hinrichs et al., 2017; Lee et al., 2017; Li et al., 2017c; Qiao et al., 2017; Zeng et al., 2017; Cho et al., 2018; Gong et al., 2018; Nguyen et al., 2018; Wang et al., 2018a; Wang et al., 2018b; Wu et al., 2018; Zhou et al., 2018; Huang et al., 2019a; Luo et al., 2019; Moon et al., 2019; Song et al., 2019; Yuan et al., 2019; Wu et al., 2019)	Stability of the insertion through multiple generations	Necessary specialized structure (physical and technical).
			Plant chemical element transporters (Wu et al., 2017;Kim et al., 2018a; Oda et al., 2018)		
			Biotic stress resistance (Vo et al., 2018; Yoo et al., 2018; Ma et al., 2019)		
			Yield and quality (Um et al., 2018; Xiao et al., 2018a; Yang et al., 2018; Wang et al., 2018c; Huang et al., 2019b)		
			Abiotic stress tolerance (Almadanim et al., 2017; Chen et al., 2018; Kwon et al., 2018; Giri et al., 2018; Li et al., 2018d; Redillas et al., 2019; Tang et al., 2019; Xia et al., 2018; Xiao et al., 2018b; Zhang et al., 2018; Deng et al., 2019; Qin et al., 2019; Zhan et al., 2019; Wang et al., 2019)		
Transposon	Random sequence insertion	51% (in 4413 families) (Kolesnik et al., 2004)	Plant development and metabolism (Ramamoorthy et al., 2018; Ke et al., 2019);	*Ac/Ds* transpose at high frequencies in rice.	
Retrotransposon	Random sequence insertion	1 insertion each 100-kb (Miyao et al., 2003)	Plant development and metabolism (Hori et al., 2016; Mieulet et al., 2016; Chen et al., 2017b)	Insertion events are more frequent in genic regions.	
			Yield and quality (Hirose et al., 2017; Imagawa et al., 2018)		
			Industrial quality (Nakata et al., 2018)		

*From the last 2 years.

### Transposon and Retrotransposon Mutagenesis

The DNA transposon activities in *Oryza sativa* and *Oryza glaberrima *are responsible for the increase of the mutation number in transposon flanking sequences in these species (Wicker et al., 2016). With the excision and reinsertion of sequences performed by transposons, the DNA repair can cause up to 10 times more mutations, mainly because transposons preferentially insert in sites close to genes, resulting in higher frequency of mutations in genic regions. In addition, the same phenomena were identified in the maize (*Zea mays*), barley (*Hordeum vulgare*) and wheat (*Triticum* ssp.) genomes, revealing a new process related to gene evolution in grasses (Wicker et al., 2016).

The transposon insertional activity has been widely applied to produce large scale mutations in rice genomes, as reviewed by Wang and collaborators (2013), which described that transposons such as *miniature Ping* (*mPing*), 607-pb and *nDart1-3* were used for rice mutant line development. Also, two components of the maize transposon system, *Activator*/*Dissociation* (*Ac*/*Ds*) and *Enhancer/Suppressor Mutator* (*En/Spm-dSpm*) have been widely applied to produce insertional mutants in rice (reviewed in Wang et al., 2013; Xuan et al., 2016). In the presence of *Ac transposase*, the *Ds *element tends to be transposed and randomly integrated into the genome (McClintock, 1950). The *Ac*/*Ds* transposable system was applied in rice to produce mutagenesis (Upadhyaya et al., 2006; Xuan et al., 2016). In generated populations, more than 70% of mutant lines showed *Ds*-independent insertions. The *Ds* insertion in *OsPS1-F* (*Photosystem 1-F subunit*) gene caused its loss of function, and *OsPS1-F* was characterized as a regulator of plant growth and development through electron transport (Ramamoorthy et al., 2018). The autonomous *Ac/Ds* element was reported to transpose with high frequency in rice somatic and germinal cells (reviewed in Kolesnik et al., 2004).

The retrotransposon insertion can affect gene function since it preferentially inserts into genic rather than intergenic regions (Miyao et al., 2003). Therefore, the retrotransposon *Tos17* insertion in the upstream region of *OsHd1* (*Heading date 1*) that altered the rice flowering time (Hori et al., 2016). The *Tos17(Hd1)* showed late flowering in short day conditions and premature flowering in long days, different from the corresponding wild-type Nipponbare.

Transposable elements played and continue to play an important role in rice evolution. The analyses of the retrotransposon mobility in 3,000 rice genomes showed that the subspecies *indica*, *japonica* and *aus/boron* originate from wild relatives that have diverged a long time ago, supporting the hypothesis that these subspecies originated from multiple domestication events. These findings demonstrate the importance of transposable elements in rice variability and evolution (Carpentier et al., 2019).

## Targeted Mutation

Physical and chemical mutagens cause random mutations, providing a limiting mutation frequency in desired/target loci. On the other hand, the genome editing systems (meganucleases – MN; zinc finger nucleases – ZFN; transcription activator-like effector nucleases – TALENS; clustered regularly interspaced short palindromic repeat – CRISPR) induce target mutations and can be used as an alternative mutagen (**Table 3**).

**Table 3:** Mutagens applied for induction of target mutations in rice.

Method	Mechanism of mutation	Mutation frequency	Mutants produced	Pros	Cons
MN	Generates short 3’ overhangs, predominantly result in larger deletions, with occasional larger insertions.	Has not been applied to rice		MNs generate deletions, which range in size from 2 to 71bp in maize.	Native MNs targeted sequences are limited; Introduction of specificities are difficult because the DNA-binding and endonuclease activities are on the same domain.
ZFN	Predominantly small deletions, but a larger proportion of insertions than TALENs.	−	Identification of safe harbor loci (Cantos et al., 2014);	ZFNs are easy to design, due the preexisting Zinc Fingers known recognition patterns.	ZFNs produce more insertions than TALENs, it was proposed as a disadvantage if engineered cereals are taken through the regulatory approval process.
			Plant development and metabolism (Jung et al., 2018);		
			Industrial quality (Jung et al., 2018);		
TALEN	Predominantly small deletions and occasionally insertions and substitution.	calli T0 (4-30%) (reviewed in Zhu et al., 2017)	Biotic stress resistance (Li et al., 2012);	TALENs bind with greater specificity than ZFNs.	Construction of the DNA recognition motif is laborious; Require a thymine at the first position.
			Industrial quality (Ma et al., 2015; Shan et al., 2015)		
			Herbicide resistance (Li et al., 2016b)		
CRISPR/Cas9	Small indels (≤10pb), often single nucleotides, inserts mostly A/T base pairs and deletions.	85%-100% (reviewed in Zhu et al., 2017)	Yield and quality (Li et al., 2016d; Li et al., 2016e; Xu et al., 2016; Zhou et al., 2016; Miao et al., 2018; Sun et al., 2018; Barman et al., 2019; Li et al., 2019; Pérez et al., 2019)	High specificity through gRNA.	Undesirable off-targets, PAM requirement for AT residues, high quantity of mismatches tolerance.
			Plant development and metabolism (Li et al., 2017c; Cheng et al., 2019; Lee et al., 2019b)		
			Nutritional quality (Sun et al., 2017; Songmei et al., 2019; Yang et al., 2019)		
			Biotic stress resistance (Zhou et al., 2015; Wang et al., 2016; Macovei et al., 2018; Li et al., 2019)		
			Abiotic stress tolerance (Xu et al., 2014; Sun et al., 2016; Zhang et al., 2019)		
CRISPR/Cpf1 (Cas12)	Generate 5’ overhangs which predominantly result in deletions.	47.2% (Endo et al., 2016)	Plant development and metabolism (Hu et al., 2017; Tang et al., 2017; Wang et al., 2017; Yin et al., 2017)	High specificity through crRNA and less frequency of off-targets.	PAM requirement for GC residues.

The targeted genome editing is mediated by different nucleases which introduce DNA double-strand breaks (DSBs). Those can promote single or double cuts, stimulating the cellular DNA repair mechanisms (reviewed in Zhu et al., 2017). Cells present two different repair systems, homologous recombination (HR) and nonhomologous end-joining (NHEJ) (Bortesi and Fischer, 2015). According to the repair system used, different modifications will be obtained, promoting genetic variability generation from DBSs induced by nucleases.

### DNA Double-Strand Break Repair by Homologous Recombination

During meiosis, Spo11-induced DNA double-strand breaks (DSBs) are repaired exclusively by homologous recombination (HR) using allelic chromosome sequence as a template. However, in somatic cells, HR acts less frequently, being its efficiency influenced by the availability of homologous sequence. In this case, homologous sequences present in the same chromosome, in genome or sister chromatid after replication can be used (Puchta, 2005).

The HR repair system can be classified as conservative and non-conservative. In non-conservative systems, the genetic material is lost, while in conservative systems losses do not occur. The single-strand annealing (SSA) is a non-conservative mechanism and depends on the proximal homologous sequence. After DSB occurrence, a single strand degradation (resect) occurs in both broken sites, until the homologous sequences are annealed. The pending single strands are degraded, or the gaps are filled, followed by the strand connecting (reviewed in Steinert et al., 2016).

Synthesis-dependent strand annealing (SDSA) and the double-strand break repair (DSBR) are conservative mechanisms. Overall, DSBR occurs in meiosis and can result in crossing over between homologous chromosomes, while SDSA works mostly by repairing DSBs in somatic cells. In SDSA, after a DSB, the homologous sequence is copied at the broken site and no loss of genetic information occurs. The breakpoints are degraded to expose the 3’ end of the single strand. One of the single strands with 3’ free-end invades the double stranded homologous sequence, being elongated based on the invaded template strand. After, the elongated single strand is released and can be reannealed with the homologous single strand (reviewed in Steinert et al., 2016).

HR can occur using as template the endogenous DNA (homologous to DSB) or an exogenous sequence displaying homology regions. In this case, together with the editing system used, a plasmid containing the donor DNA or the PCR product itself corresponding to the desired sequence (Song and Stieger, 2017) should be introduced into the plant. The donor DNA should have the 5’ and 3’ homologous borders to the target DNA, and the central region containing the sequence to be edited (nucleotide exchange) or sequence to be inserted.

### DNA Double-Strand Break Repair by Nonhomologous End-Joining

In most plant and animal genomes, the HR repair mechanism is less frequent than the nonhomologous end-joining (NHEJ) mechanism (Bortesi and Fischer, 2015). Differently from HR, NHEJ does not require a homologous sequence for DNA repair and is therefore prone to errors (Manova and Gruszka, 2015). The NHEJ mechanism is subdivided into cNHEJ and aNHEJ. The cNHEJ mechanism begins with the recognition of DSB by the heterodimer Ku70/Ku80, which forms a ring that binds at both ends and recruit DNA-PKcs kinase, preventing the degradation (Mannuss et al., 2012; Nishizawa-Yokoi et al., 2012; Manova and Gruszka, 2015), which results in minimal change in genetic information (Puchta and Fauser, 2014). The lesion is processed by different proteins aiming at the restoring, respectively, of the phosphate and hydroxyl groups in the DSB 5’ and 3’ ends, and finally the DNA binding (Rulten et al., 2011; Manova and Gruszka, 2015).

In aNHEJ processing, degradation of the final 3’ end occurs. The annealing of the two single strands leads to the formation of junctions where few complementary nucleotides are present. Generally, terminal regions are trimmed, re-joining occurs and microhomologies are exposed. It is possible that the genetic information at the junction site is lost. In this sense, aNHEJ process is considered a highly mutagenic repair mechanism (Puchta and Fauser, 2014; Manova and Gruszka, 2015). Therefore, for the targeted mutation, one could expect a repair by NHEJ when the editing aims at silencing, but repair by HR would be expected when the aim is proper editing (with DNA donor).

### Meganuclease

**Figure 4:**
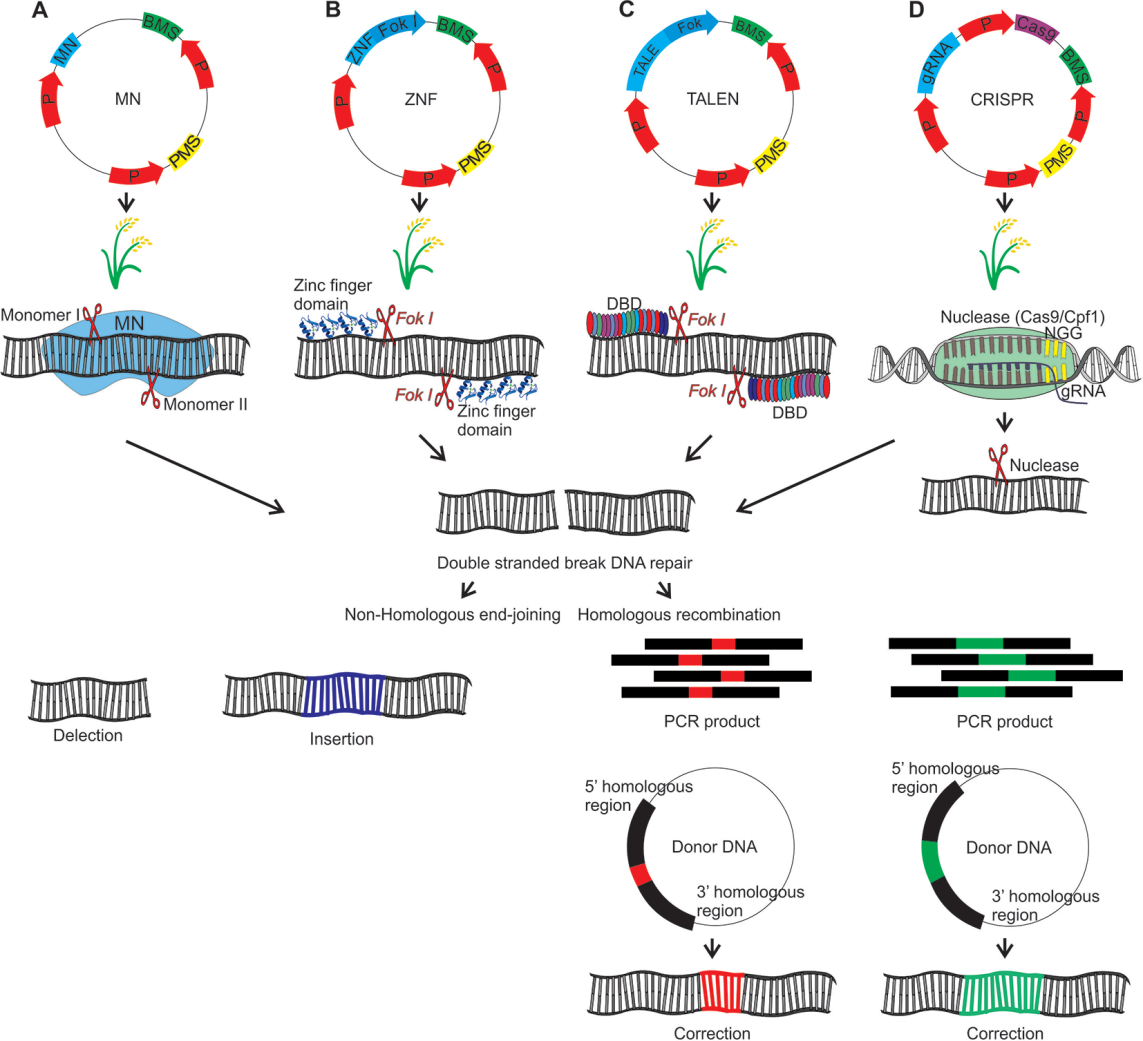
Genome editing using MN, ZFN, TALEN and CRISPR strategies which result in the DNA double strand break that will be repaired by homologous recombination (HR) or nonhomologous end-joining (NHEJ). HR requires the introduction of a donor DNA plasmid or a PCR product corresponding to the donor DNA. For HR should be inserted a donor DNA together with the editing system. In this hypothetical scheme a gene associated with seed yield in rice will be edited. **(A)** Meganuclease (MN): The DNA binding domain of the meganucleases are engineered to recognize specific target sequences. **(B)** Zinc finger nuclease (ZFN): Zinc finger transcription factors present the C2H2 motif with DNA recognition capability. The ZFN system function requires the fusion of ZF motifs with the restriction enzyme *Fok I*. *Fok I* presents a DNA binding domain that recognizes a 5-’GGATG-3’ sequence and a nonspecific DNA cleavage domain. For the zinc finger nuclease (ZFN) system, *Fok I* was engineered, and the DNA binding domain was removed. In this sense, the *Fok I* nonspecific DNA cleavage domain was used to construct a hybrid nuclease, through binding to the ZF binding domain. **(C)** Transcription activator-like effector nucleases (TALEN): The TALE DNA binding domain is formed by monomers, each monomer recognizing only one nucleotide. Each monomer is composed by 34 amino acids, that are repeated in the other monomers, except the hypervariable amino acids located at positions 12 and 13 of each monomer, which are determinants for binding to a specific nucleotide. These amino acids are known as repeat variable di-residues (RVDs). The nonspecific *Fok I* DNA cleavage domain, was used to construct a hybrid nuclease, through the binding with TALE DNA binding domain, forming the TALEN system. **(D)** Clustered regularly interspaced short palindromic repeats (CRISPR): In this system, a guide RNA (gRNA) (crRNA + tracrRNA) directs a restriction enzyme to a specific DNA sequence that will be cleaved. P, promoter; PMS, Plant marker selection; BMS, Bacterial marker selection; DBD, DNA binding domain. The plasmid introduction into rice plants can be performed directly by electroporation and biobalistic methods or indirectly* via Agrobacterium tumefaciens*. In this last case it is necessary to add the right and left borders in the plasmid containing the editing system.

Meganuclease (MNs), also called homing endonucleases (HEases), are site-specific endonucleases (they recognize and cleave long sequences - 14 to 40 bp - with high specificity). These can be manipulated through different strategies to increase the number of target sites, allowing its use as a tool for genome editing (Arnould et al., 2011; Guha et al., 2017). MNs can induce DSBs, which will be repaired by HR (with donor DNA) or NHEJ, an error-prone process due to insertions or deletions at the cleavage site (Arnould et al., 2011) (**Figure 4A**). The MNs system has been used efficiently in *Arabidopsis*, cotton and maize (reviewed in Weeks et al., 2016). To our knowledge, there are no studies applying MN in rice mutagenesis, however these studies in *Arabidopsis* and other species indicate its potential as a tool for rice, aiming at targeted mutation.

### Zinc Finger Nucleases

Zinc finger nucleases (ZFN) are restriction enzymes generated by the fusion of a non-specific DNA cleavage domain (*Fok I*) with site specific DNA binding zinc finger proteins (ZFPs) (Kim et al., 1996; Guo et al., 2010). The endonuclease *Fok I* (*Flavobacterium okeanokoites*) shows a DNA binding N-terminal domain and a C-terminal domain with non-specific DNA cleavage activity. In this sense, that enzyme can be engineered to cleave a specific DNA site through the binding to other domains which recognize specific sequences (Kim et al., 1996). The modular structure of C_2_H_2 _zinc finger motifs and their independent recognition capability make this structure suitable for the engineered ZNP development with specificity for new sequences. Each motif recognizes 3 or 4 nucleotides on the α-helix and the tandem ZF modifications enable the specific recognition of DNA sequences (Liu et al., 1997; Beerli and Barbas, 2002; Guo et al., 2010). ZFNs are applied in genome editing through the introduction of DSBs on specific sites in the genomic DNA. Thus, target genes can be interrupted by mutagenesis caused by NHEJ repair or modified *via* HR if a homologous DNA template is provided (Guo et al., 2010) (**Figure 4B**).

ZFN has been reported as efficient for genome editing and can be used as a new rice breeding technology for variety development (Cantos et al., 2014). Recently, to unveil the *SSIVa* (*Starch Synthase IVa*) gene function, ZFNs targeting the coding region were used to induce DSBs (Jung et al., 2018). Transgenic plants presented premature stop codons and substitution events, leading to inactivation of the *SSIVa* gene, low starch content and dwarf phenotypes.

### Transcription Activator-Like Effector Nucleases

Transcription activator-like effector (TALE) proteins occur naturally in the *Xanthomonas* genus of phytopathogenic bacteria (reviewed in Nemudryi et al., 2014). TALE contains a DNA binding domain formed by monomers of repeated 34 amino acids, each domain recognizing unique base pairs. The TALE specificity is determined by two hypervariable amino acids, located in the 12^th^ and 13^th^ position of the monomer, which are known as repeat-variable di-residues (RVDs). Moreover, TALE contains a nuclear signal localization and a domain that activates the gene target transcription (reviewed in Nemudryi et al., 2014). RVDs can be engineered to generate DNA binding proteins which can be applied to specific site genome editing. The independent sequence of *Fok I* nuclease acts as a nuclease site specific for the TALEN genome editing system (fusions of an engineered TALE repeat array to the non-specific *FokI* endonuclease domain) when target sites are recognized by different TALEs (reviewed in Ma and Liu, 2015).

TALENs can be applied to introduce genome DSBs. The target DSBs are repaired by NHEJ, resulting in mutation by insertion or deletion in the broken site. DSBs can also be repaired by HR, with a homologous donor DNA template, which is used to introduce single nucleotides or major insertion changes (Reyon et al., 2013) (**Figure 4C**). In rice, TALENs were reported to produce a large rate of heritable mutations, been transmitted from T1 to T2 at Mendelian proportions. Also, 81% of the mutations affected multiple bases, being 70% of the mutations caused by deletions (Zhang et al., 2016). Aspects regarding mutations caused by TALENs in rice are well described (Zhu et al., 2017). In callus (T0), the frequency of mutations reported was 4-30%, generating frequently chimeric, heterozygous, bi-allelic and in some cases homozygous plants. TALENs have been used to generate mutations in many different genes in rice in order to improve traits such as industrial quality, biotic stress resistance and abiotic stress tolerance (**Table 3**).

Although TALENs comprehend a laborious technique in the construction of the DNA recognition motif, the reported studies demonstrate that this tool can be applied directly for rice improvement.

### Clustered Regularly Interspaced Short Palindromic Repeat

Undoubtedly, most of the recent rice studies involve the use of the clustered regularly interspaced short palindromic repeat (CRISPR) system, which promotes a higher frequency of mutations compared to TALEN. The major features of mutations caused by CRISPR/Cas9 (CRISPR-associated protein) in rice are well described (Zhu et al., 2017). The frequency of mutations induced by the CRISPR/Cas9 system in rice ranged from 85% to 100%. In addition, using a specific gRNA, bi-allelic mutants were reported in T0, representing up to 100% of the mutations while homozygous individuals were obtained in frequencies of up to 50%. A way to increase the frequency of homozygous mutations is to use gRNAs targeting the same gene in different regions. In general, mutations induced by TALEN and CRISPR/Cas9 systems in rice are typically insertions or deletions of one or hundreds of base pairs, with only 4% single nucleotide mutations reported (Zhu et al., 2017).

The CRISPR mechanism is part of the adaptive immune system of bacteria and archaea against invading nucleic acids such as viruses. This is accomplished by cleaving the invader DNA in specific sequences. The immunity is acquired by the invader DNA insertion (spacers) between two adjacent repeats in the proximal end of the CRISPR locus. When the nucleic acid invasion occurs, the CRISPR locus and the spacers are transcribed, these are processed in a small interfering CRISPR RNA (crRNA), that combines with transactivating CRISPR RNA (tracrRNA) for nuclease Cas activation (gene associated to CRISPR) directed to the nucleic acid invader. This mechanism is able to perform cleavage of invader DNA homologous sequence (spacer) (Barrangou et al., 2007, Bortesi and Fisher, 2015). The presence of an adjacent protospacer motif (PAM) downstream of the invader DNA showing a 5′-NGG-3′ or a 5’-NAG-3’ sequence is a requirement for cleavage (Gasiunas et al., 2012, reviewed in Bortesi and Fisher, 2015; reviewed in Zhu et al., 2017). NGG-PAM is abundant in plant genomes and rice presents an NGG-PAM each 9.8 bp (reviewed in Zhu et al., 2017). This system enabled the development of the latest innovative genome-editing technology based on engineered nucleases guided by RNA. Fusing the crRNA with tracrRNA to form a single synthetic guiding RNA (gRNA) simplified the process (Jinek et al., 2012).

The DSB repair process is fundamental for the CRISPR system efficiency. The repair types include HR and NHEJ. In the NHEJ repair mechanism, small deletions, but rarely insertions are introduced. Loss of function mutations resulting in sequence changes causing alterations in the reading phase or changes in amino acids can be found in the gene final product (reviewed in Choudhary et al., 2017) (**Figure 4D**). In DSB repair by HR mechanism, a donor DNA template is used as a source of DNA information which is copied for the broken chromosome to restore its integrity. The donor DNA variations are copied by HR in the chromosome. In this case, it is necessary to introduce in the target organism the Cas, gRNA and also the donor DNA.

#### Crispr/Cas9

Different nucleases can be associated with the CRISPR system. The CRISPR/Cas system can be classified as type I, type II and type III, according to the presence of the Cas3, Cas9 and Cas10 proteins, respectively (Makarova et al., 2011). Type II is the most widely used genome editing system (reviewed by Bortesi and Fisher, 2015). CRISPR system type II from *Streptococcus pyogenes* has been adapted to induce specific DSBs and editing the genome in target regions. The most simply and widely applied method requires introduction and expression of two components in a target organism, the Cas9 coding gene and a gRNA (crRNA + tracrRNA) homologous to the target sequence. Cas9 can be directed to any DNA sequence, being the specificity given by the gRNA sequence corresponding to the target DNA sequence (Sander and Joung, 2014). The most common mutations induced by Cas9 in rice are small indels (≤10pb), often single nucleotides, and inserts which are mostly A/T base pairs (Zhu et al., 2017).

Important features related to gRNA for CRISPR/Cas9 system need to be pointed out. The gRNA design must consider that Cas9 tolerates up to three mismatches in the gRNA-DNA pairing region, and mismatches close to the NGG-PAM motif significantly reduce the Cas9 affinity for the target site. Thus, mismatches can only be tolerated in the distal position of the protospacer. Due to mismatches between the gRNA and the target site, non-target (off-target) sites are more common in CRISPR/Cas9 system. Mismatches within the first 12 bp near to PAM generally nullify DNA cleavage in rice while mismatches smaller than 12 bp in regions close to PAM, increase the chance of off-targets in rice. gRNAs with high GC content achieve higher editing efficiency in rice if used in CRISPR/Cas9 system. It is estimated that specific gRNAs can be designed for 89.6% of the annotated rice transcripts (Zhu et al., 2017).

Some parameters have been identified which affect the frequency of CRISPR/Cas9 mediated mutagenesis in rice calli (Mikami et al., 2015). A positive correlation regarding Cas9 expression mutation frequency was detected, extending the callus culture period expressing Cas9 and gRNA, there is an increase in the rate of mutated/non-mutated cells and in the variety of mutations obtained. Nevertheless, double DSBs can be induced with the aim of deleting fragments. For CRISPR/Cas9 system, deletions (200bp) and greater deletions (357-761bp) were obtained in frequencies of 10% and 4-45% in T0 plants from rice protoplasts, respectively (Zhu et al., 2017). Also, deletions of a chromosomal segment (115-250Kb) were detected in frequencies ranging from 16 to 25%.

CRISPR/Cas9 system has been used to mutagenize rice genes for a diverse range of purposes, mainly for important agricultural traits (**Table 3**). Improvements in the technique has been obtained in the last few years. In this sense, the rice multiple gene edition system using CRISPR/Cas9 was developed (Wang et al., 2015). This multiplex system was obtained providing a co-expression of the Cas9 protein and many gRNAs, being able to edit different genes simultaneously. The multiplex CRISPR/Cas9 system was applied to edit four genes, *OsGS3* (*Grain Size 3*), *OsGW2* (*Grain Width 2*), *OsGW5* (*Grain Width 5*) and *OsTGW6* (*Thousand-Grain Weight 6*) obtaining plants with higher grain weight (Xu et al., 2016). Also, CRISPR/Cas9 has been applied to targeting T-DNA insertion in rice genomes (Lee et al., 2019a). Formerly considered a random insertion, nowadays this technology has been improved by CRISPR/Cas9.

#### Crispr/Cpf1

A new CRISPR system from *Prevotella* and *Francisella* 1 (Cpf1 or Cas12a) was reported to efficiently produce directed mutagenesis in rice and tobacco (*Nicotiana tabacum*) (Endo et al., 2016). Cpf1 enzymes belong to the family V of CRISPR nucleases that include both endoribonuclease and endodeoxyribonuclease activities, favoring its use in the CRISPR-RNAs (crRNAS) process and in the introduction of DSBs into DNA, respectively (reviewed in Begemann et al., 2017). In addition, Cpf1 nucleases have been shown to have low off-target editing rates compared to Cas9 nucleases (Begemann et al., 2017; Tang et al., 2018). However, off-targets were identified associated with Cpf1 activity in rice due to mismatches at 11^th^ nucleotide from PAM, demonstrating that Cpf1 also tolerates mismatches (Endo et al., 2016).

The system consists of a new guide RNA endonuclease with two different traits when compared to Cas9: Cpf1 utilizes a PAM motif rich in thymine, while Cas9 uses a PAM motif rich in guanine, in this way Cpf1 can be applied as a sequence specific nuclease for rich AT targets. Also, Cpf1 generates DNA ends with a 5’ overhang, whereas Cas9 creates blunt DNA ends after cleavage. Also, it was reported that crRNA and tracRNA are required for the Cas9 action, however Cpf1 needs only a 44-nucleotide crRNA localized 5’ directing after the repeated sequence and spacer to guide the Cpf1 action (Zetsche et al., 2015). In addition, it was reported that the CRISPR/Cpf1 system performs editing and transcriptional repression in rice (Tang et al., 2017). Regarding multiplex targets, a study targeting up to four genes was also reported in rice, (Wang et al., 2017). Studies using Cpf1 from *Fransicella novicida* (FnCpf1) and *Lachnospiraceae bacterium* ND2006 (LbCpf1) have shown that Cpf1 are efficient in DNA insertion through HR repair when used together with a crRNA and DNA donor in rice (Begemann et al., 2017). The mutation frequency obtained for different loci in rice was reported to reach 47.2% (Endo et al., 2016). In addition, the induced mutations were mostly deletions, monoallelic and bi-allelic (Endo et al., 2016; Xu et al., 2017b). Diverse studies demonstrate the application of CRISPR/Cpf1 system in rice, mainly for aspects of plant development and metabolism (**Table 3**).

#### Peculiarities of the CRISPR/Cas System

Recently, the CRISPR-GE software was used for gRNA design, target site prediction, primer design for construction of gRNA expression cassettes and target site amplification (Xie et al., 2017). Also, it has been used for the detection of mutants from genomic PCR amplicon chromatograms containing the target sites and for the download of genomic sequences from reference genomes in plants and other organisms. Also, it is suitable for the choice of appropriate target site for Cas9 or Cpf1 nucleases.

One of the problems of CRISPR mediated editing is the off-targets. In this sense, CRISPR-GE helps in reducing the chances of off-target editing because it allows the selection of highly specific gRNAs. Another way of decreasing off-target chances, is the use of Cas9 from different microorganisms that recognize larger PAM sequences. Importantly, Tang et al. (2018) identified off-targets related to Cas9 activity in T0 rice while no off-target was related to Cpf1 activity. However, in T1 plants no off-targets, neither related to Cas9 nor to Cpf1 were identified. These data are important and demonstrate that it is possible to use Cas9 and Cpf1 in certain breeding applications across several generations (Tang et al., 2018). Off-targets are not always a problem, researchers have used off-targets as a way to edit more than one gene with only a single gRNA (Endo et al., 2015).

Due to the difference in the effect of mutations caused by the same editing system on rice genes, factors that influence the DSB repair, which is determined by some gene traits, independent of the nuclease used, have been suggested (Zhu et al., 2017). Overall, these observations demonstrate the complexity of mutagenesis techniques and pave the way for further understanding of gene functions and the development of superior rice plants.

## Methods for Mutation Detection

**Figure 5:**
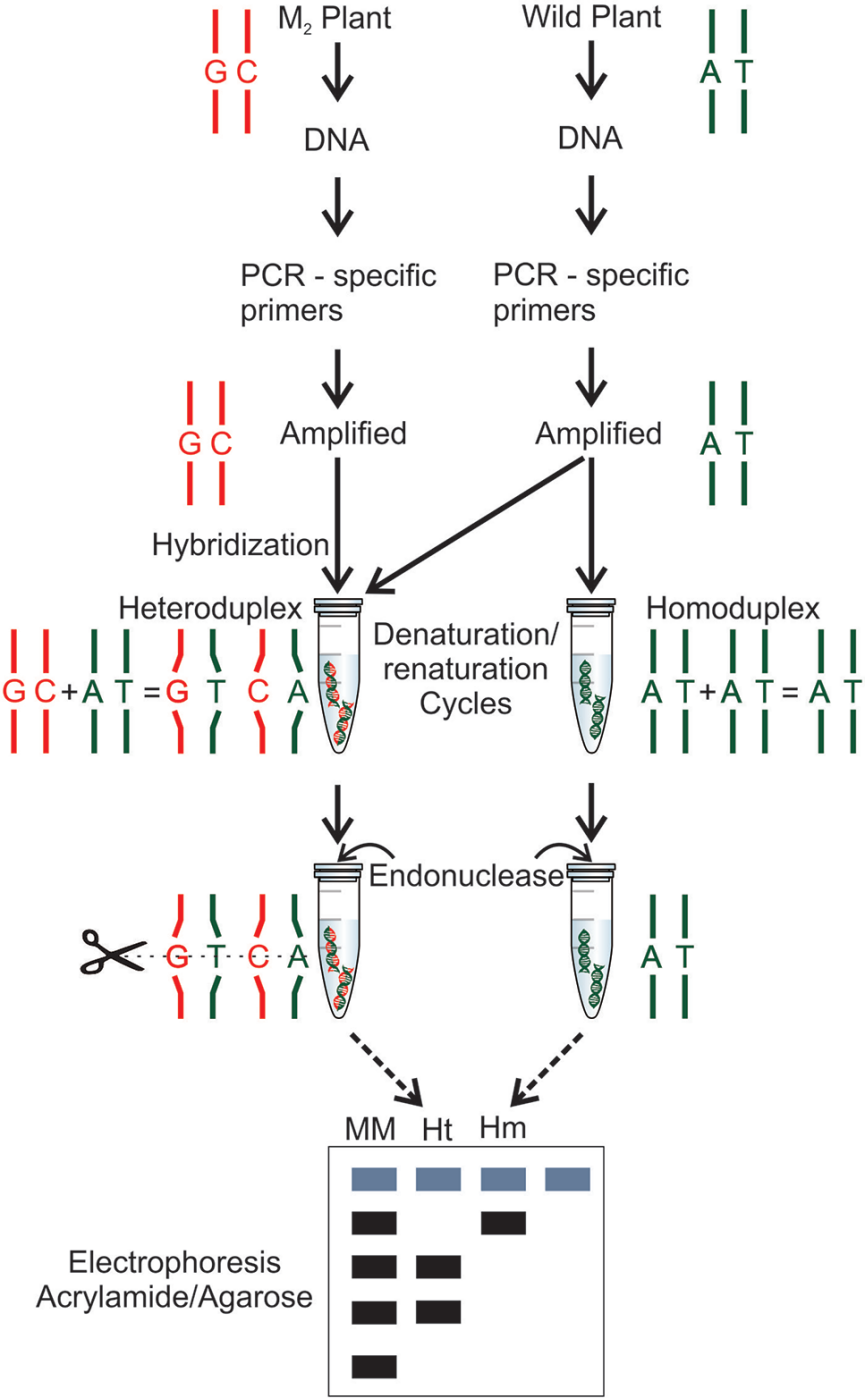
Mutation detection by TILLING technique (Till et al., 2003). The wild-type and mutant DNAs are subjected to a polymerase chain reaction (PCR) using gene specific primers. The amplified DNA is combined in the same tube, amplified mutant DNA and amplified wild-type DNA. In a second tube, the wild-type DNA is added two times. Both tubes are subjected to denaturation and renaturation cycles, forming, in a mutant case, an heteroduplex, a hybrid double stranded with a mutant strand and a wild-type strand, and in a wild-type case, a homoduplex is formed with a wild-type double stranded DNA. After, an endonuclease (*CEL I*) treatment is performed in both DNAs (hybrid and no hybrid), the nuclease is able to find and recognize the mismatch pairs in the hybrid DNA and cleave the double strand DNA. Both DNAs are subjected to electrophoresis using a molecular marker (MM), the heteroduplex (Ht) digested by the endonuclease and the undigested homoduplex (Hm).

Many different methods for mutation detection have been applied and developed for rice (**Table 4**). TILLING (Targeting Induced Local Lesions IN Genomes) is one of the most widely used techniques in reverse genetics for detection of point mutations (**Figure 5**). First, the technique was developed for the detection of point mutations generated by chemical mutagens (McCallum et al., 2000). However, it has been used in mutant rice populations generated with EMS (Till et al., 2007; Serrat et al., 2014), MNU (Suzuki et al., 2008), combination of MNU and SA (Till et al., 2007) and γ-rays (Cho et al., 2010; Chun et al., 2012). Furthermore, it has been used in polyploid organisms such as wheat (Sestili et al., 2010) and autotetraploid Arabidopsis (Tsai et al., 2013) allowing to be applied in rice polyploidy also. TILLING by sequence is an application of the new generation sequencing (NGS) and bioinformatics in mutant populations (Kumar et al., 2017). TILLING by sequence was applied in rice mutant populations obtained with MNU and SA treatment (Tsai et al., 2011; Kim and Tai, 2014). TILLING-high-resolution melting (HRM) detect mutations in target genes using PCR, a process monitored by a DNA-binding dye. With the DNA double strand renaturation, the fluorescence increases resulting in a melting curve (reviewed in Taheri et al., 2017). In rice, TILLING-HRM was applied to detect DNA changes induced by γ-rays (Li et al., 2018b). Since the generation of exon mutations is quite interesting for gene functional studies, a technique using exome capture associated with NGS was applied in rice TILLING population obtained by EMS treatment (Henry et al., 2014). Through EcoTILLING, natural variations in the rice genome have been identified (Vaughn and Bennetzen, 2013). EcoTILLING is also a reverse genetics technique and apply the same principles of TILLING but in natural populations rather than in an induced mutant population (reviewed in Barkley and Wang, 2008). Eco-TILLING by sequence was used to identify polymorphisms in genes coding for starch synthases potentially associated with the increase of resistant starch and the reduction of the hydrolysis index (Raja et al., 2017).

**Table 4:** Mutation detection methods applied in rice.

Method	Type of mutagenesis applied	Type of mutation detected	Pros	Cons	Reference
TILLING	EMS, MNU, SA, γ-ray	SNPs	High sensitivity; Provides the approximate location of the induced mutation; Detect induced and naturally occurring homozygous and heterozygous SNPs; Suitable for polyploids.	Require celery *CEL I* endonuclease for the mismatch detection; False negatives and positives (are low but exist).	McCallum et al., 2000; Till et al., 2003; reviewed in Barkley and Wang, 2008 and Taheri et al., 2017
TILLING-NGS	MNU, SA	SNPs	No require enzymatic digestion; High throughput; Time saving; Efficient in polyploids; Mutation detection in pools deeper than eight individuals.	Expensive; Needs multi-dimensional pooling; Can incorrectly identify DNA bases with high frequency which is not easy to identify due the amount of data produced; It is laborious to process, storage and analyze the data.	Kumar et al., 2017; reviewed in Taheri et al., 2017
TILLING-HRM	γ-ray	SNPs, indels	No require enzymatic digestion; High sensitivity; Time and cost saving.	Depends on good PCR instruments and dyes; Needs multi-dimensional pooling; More difficult to detect indels than substitutions; Sensitivity limited to amplicons <450 bp.	Li et al., 2018b; reviewed in Taheri et al., 2017
Exome capture	EMS	SNPs, indels	Large-scale mutation discovery; High-throughput; Cost-effective; Applicable in polyploids.	It is laborious to process, storage and analyze the data; Need transcriptome assembly in cases a reference genome is not available.	Henry et al., 2014; reviewed in Taheri et al., 2017
Eco-TILLING	Natural mutations	SNPs	Provides the approximate location within a few base pairs of the induced mutation; Detect induced and naturally occurring homozygous and heterozygous SNPs;	Require *CEL I* endonuclease mismatch detection celery; False negatives and positives (are low but do exist).	reviewed in Barkley and Wang, 2008
MutMap	EMS	SNPs	Minimizes the number of crosses in crop species and required mutant F2 progeny.	Not suitable for plants without reference genome sequence (now improved by MutMap-GAP).	Abe et al., 2012; Takagi et al., 2013; Taheri et al., 2017
CRISPR-S	CRISPR/Cas9	−	Enable a PCR-free, phenotype-based identification of genome-edited T0 plants, and a subsequent selection of transgene-free T1 plants.	Require a RNAi expression element incorporated into the CRISPR/Cas9.	Lu et al., 2017
PCR-based	CRISPR/Cas9	short indels (± 1pb)	Accurately identify indel sizes down to ± 1 bp	Efficiency is affected by target sequence; Applicable only for mutation upstream to PAM	Biswas et al., 2019
Amplicon labelingbased	CRISPR/Cas9	short indels (± 1pb)	Accurately identify indel sizes down to ± 1 bp	Sometimes could not detected the exact nucleotide change needing sequencing to confirm.	Biswas et al., 2019

**Figure 6:**
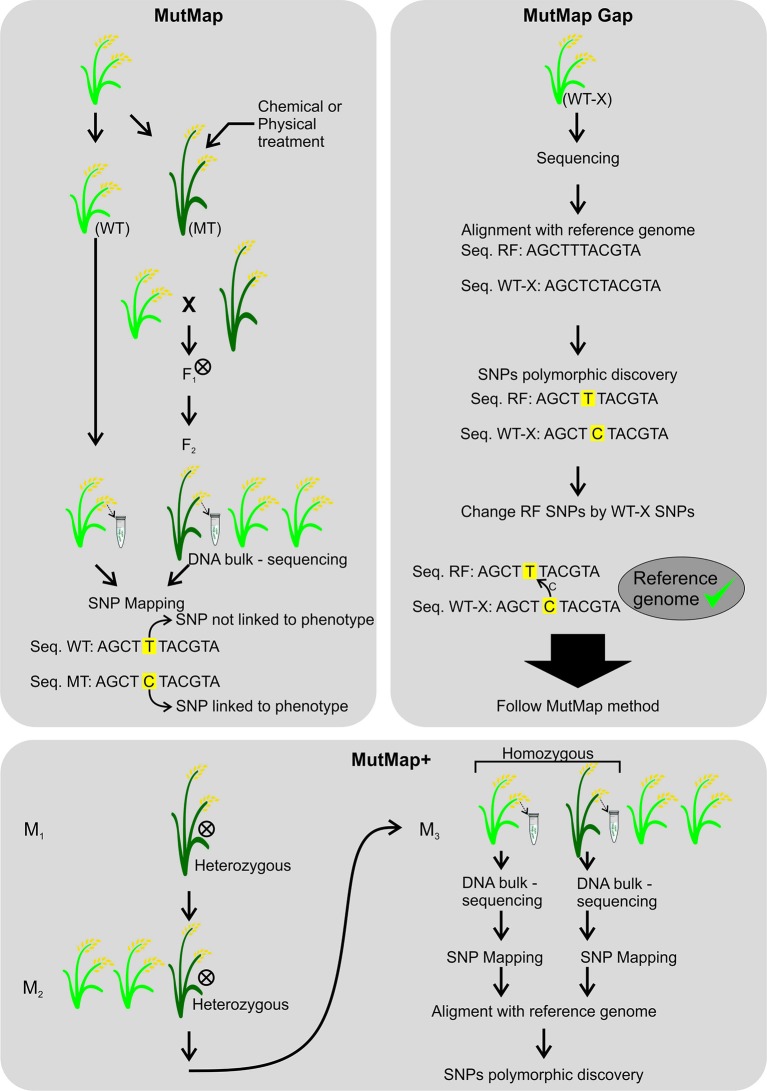
MutMap (Abe et al., 2012), MutMap Gap (Takagi et al., 2013) and MutMap+ (Fekih et al., 2013) techniques. MutMap was developed to identify mutations with polymorphic single nucleotide (SNPs) between a mutant and wild-type genotypes. Both plants are crossed and the F_1_ generation is self-pollinated (⊗). DNAs are extracted from the wild-type and mutant F_2_ to perform DNA sequencing followed by SNP mapping to find mutations linked to the phenotype. The MutMap Gap was developed when there is no available sequence of the desired genotype for mutation. The desired genotype (WT-X) is sequenced and aligned to the reference genome (a sequenced genotype) and the polymorphic SNPs are found and changed between WT-X and the reference genome to produce a desired reference genome. After the MutMap is followed. The MutMap+ was developed to avoid crosses between wild-type and mutant genotypes. The heterozygous plants in M_1_ and M_2_ generation are self-pollinated (⊗) and the homozygous M_3_ plants are subjected to DNA sequencing for SNP mapping. After, an alignment with a reference genome is performed in order to detect phenotype associated polymorphic SNPs.

MutMap (Abe et al. (2012) arose based on the multigenic control of most of the agronomic traits. It allows the rapid identification of causal nucleotide changes by whole genome resequencing of pooled DNA of mutant F_2_ progeny derived from crosses made between candidate mutants and the parental line, generally using SNP markers (**Figure 6**). The mutant loci responsible for the dwarf phenotype and pale leaves were identified through MutMap. MutMap-Gap technique, also proposed in rice, is an alternative for when there is much difference between the reference genome and the mutant for a given region (Takagi et al., 2013) (**Figure 6**). In MutMap+ (Fekih et al., 2013), artificial crosses are not necessary between the rice mutant and wild-type plants, the causal mutations are identified by comparing SNP frequencies of bulked DNA samples of mutant and wild-type progeny of M_3_ generation derived from selfing an M_2_ heterozygous individual (**Figure 6**).

A strategy on edited genome identification in rice was proposed by Lu et al. (2017). The CRISPR-S involves an incorporation of a RNAi in the CRISPR/Cas9 vector, in this way the presence and activity of the T-DNA on transgenic plants can be monitored based on RNAi. Also, two new methods for identification of rice CRISPR/Cas9 induced mutations were reported (Biswas et al., 2019). First, in a PCR-based method, targets are amplified using two pairs of primers for each target locus and visualized on gel electrophoresis. The second, an amplicon labeling-based method, targets are amplified using tri-primers (one being a universal 6-FAM 5′-labelled) and detected by DNA capillary electrophoresis (Biswas et al., 2019). Also, several databases for mutant identification have been developed, for both chemical and physical induced mutagenesis, as well as for insertional mutants. Robust database lists have been reported (Wei et al., 2013; Yang et al., 2013).

## Successful Direct Application of Mutants in Agriculture

### The Promise of Synthetic Biology

Besides the application in functional genomics, the insertion of T-DNA has direct application in the development of new genotypes, such as Golden rice. Firstly, the Golden rice was developed by the insertion of the T-DNA composed by the genes *phytoene synthase* and *lycopene cyclase* (from *Narcissus pseudonarcissus*) and *carotene desaturase* (from *Erwinia uredovora*) aiming beta carotene synthesis, precursor of vitamin A (Ye et al., 2000). Afterwards, with the advancement of functional genomic studies, Golden rice 2 was developed by the insertion of a T-DNA containing *phytoene synthase* (from *Zea mays*) and *carotene desaturase* (from *Erwinia uredovora*) (Paine et al., 2005). Golden rice 2 accumulates more carotenoids compared to Golden rice being a more promising source of vitamin A. Golden Rice development may have a high impact in mitigating the problem of vitamin A deficiency, although it has only been released in some countries such as Australia, Canada, New Zealand and the United States (ISAAA, 2019). In addition to Golden rice, other rice genotypes have been developed through the insertion of T-DNA and have been already released in some countries with a positive impact on agriculture (ISAAA, 2019).

### Imidazolinone Herbicide-Resistant Rice Cultivars

Weedy red rice is one of the major problems in rice cultivated areas, decreasing grain yield, increasing the production costs and depreciating the harvested product. Weedy red rice and cultivated rice belong to the same species (*Oryza sativa *L.), therefore, the selective herbicides applied for the control of invasive plants are not efficient in red rice removal. In 2002, the U.S. Louisiana State University Agriculture Center (LSU AgCenter) developed the commercial CL 121 and CL 141 genotypes resistant to imidazolinone (IMI-rice). These genotypes were developed from a mutant line, 93-AS3510, which was obtained though EMS treatment. After, using similar approaches, the PWC16 was developed and originated the CL 161, a high productive genotype. The PUITA INTA CL genotype was developed in Argentina, using a different genetic background from the one obtained in the U.S., but also originating from artificial mutation (reviewed by Sudianto et al., 2013).

IMI rice genotypes show a point mutation in the gene which encodes for the acetolactate synthase enzyme or ALS, also known as acetohydroxy acid synthase or AHAS. ALS is responsible for branched chain amino acid biosynthesis (valine, leucine and isoleucine). IMI herbicides act as no competitive inhibitors of ALS enzyme and prevent amino acid production, killing plants due to their inability of synthesizing proteins. The first mutant line, 93-AS3510, showed a codon replacement GGG by GAG in the position 654 (domain E of the ALS), resulting in an amino acid change from glycine to glutamic acid (gly654glu). In the second mutant line, PWC16, the codon AGT was replaced by AAT in the position 653 (domain E of the ALS) changing serine to asparagine (ser653asn). In the PUITA INTA CL genotype, changes in the C domain of the ALS, causing the conversion of GCG to ACG in the position 122, resulting in alanine to threonine (ala122thr) switches, were identified (Roso et al., 2010). The ALS gly654glu, ser653asn and ala122thr mutations make the enzyme insensitive to IMI, resulting in an herbicide resistant rice (reviewed in Sudianto et al., 2013).

From the first obtained mutants, new IMI rice genotypes were developed in many different Countries. In addition to the U.S., IMI rice genotypes are cultivated in North America, Nicaragua, Panama, Colombia, Brazil, Costa Rica, Uruguay, Argentina, Paraguay, Bolivia, Dominican Republic and Honduras. Also, it is cultivated in Malaysia and Italy (reviewed in Sudianto et al., 2013; reviewed in Merotto et al., 2016). In U.S. and Brazil, more than 700,000 and 600,000 ha, respectively, are cultivated with IMI rice. In Uruguay and Argentina, IMI rice occupies a cultivated area of 70,000 ha and 32,000 ha, respectively. Malaysia and Italy cultivate 95,000 and 60,000 ha, respectively (reviewed in Sudianto et al., 2013).

The IMI rice was an important event for the rice production chain, considering the losses that were caused due to red rice occurrence. IMI rice demonstrates the efficiency of mutation induction as a breeding tool, even if it is a random mutation, which required a lot of time and labor to obtain a desired change in the gene of interest.

## Conclusion and Future Prospects

**Figure 7:**
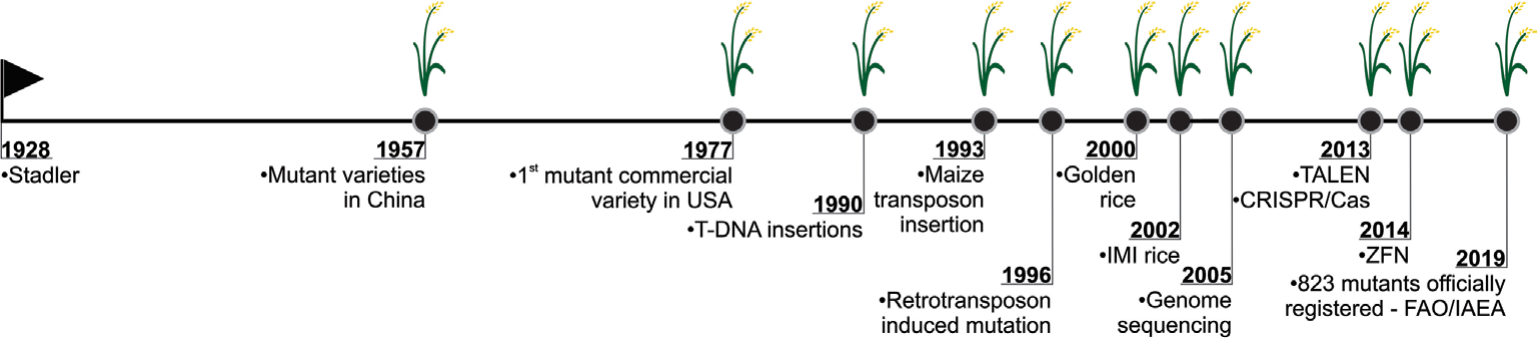
History of mutation induction in rice. Stadler’s pioneering study in inducing mutation using X-ray in barley, maize and wheat (Stadler, 1928; Stadler 1930), demonstrated the possibility in creating genetic variability through mutagenesis. It began a new era where it was possible to apply this tool in the most diverse organisms. The development of the first rice mutant in China, in 1957 (ISAAA, 2019), marks one of the important milestones in the rice mutation history. In USA (1977), the first commercial mutant variety of rice was approved (Rutger et al., 1977). After, the insertion and expression of T-DNA in rice was also reported (Raineri et al., 1990). In 1993, the autonomous element *Ac*, a component of the maize transposon system, was inserted into the rice genome (Shimamoto et al., 1993). In 1996, the use of retrotransposons to induce mutations in rice was reported (Hirochika et al., 1996). In 2002, one of the rice mutants that revolutionized agriculture was developed in USA. Imidazolinone herbicide-resistant rice cultivars are applied in invasive red rice control (reviewed in Sudianto et al., 2013). In 2002, draft genome sequences of the *japonica* Nipponbare (Goff et al., 2002) and *indica* 9311 (Yu et al., 2002) first appeared. In 2005, the map-based complete rice genome sequence was published, becoming the gold standard of crop genomes (IRGSP, 2005). Recently, genome editing technologies have been widely used in various organisms, including plants. The first techniques applied to rice mutagenesis used a non-specific nuclease (*Fok I*) associated to a DNA-specific domain, comprehend the TALEN (Transcription-like effectors nucleases) (Li et al., 2012; Shan et al., 2013) and ZFN (Zinc-finger nucleases) (Cantos et al., 2014; Jung et al., 2018). CRISPR/Cas system has been widely used to induce targeted genomic editing and has been applied in rice since 2013 (Shan et al., 2013; Jiang et al., 2013, Miao et al., 2013; Feng et al., 2013; Shan et al., 2014). Nowadays, according to FAO/IAEA records, 823 rice mutants have been officially registered since the first genotype developed in China in 1957.

The history of mutation induction began a long time ago. However, from the early days up to now, many improvements have been performed in order to increase the mutation frequency. Rice is a major crop species, and since it presents a small genome and displays synteny with other crops, many achievements in rice structural and functional genomics have been extended to other crop species. Rice was already a model since the initial findings of mutation induction to date, this includes more than 90 years of research (**Figure 7**). Much has been gained agronomically through the increase in rice variability. Gene functional characterization, development of new cultivars from mutants and even genetically engineered mutant genotypes carrying several insertions of DNA sequences were obtained through mutagenesis. Nowadays, more advanced tools such as CRISPR/Cas can be applied and further improve rice and other species. The valuable studies developed by researchers around the world have boosted science and agriculture, and every day new developments are being reported concerning mutation induction mechanisms. In addition, unveiling the genetic mechanisms of DNA damage repair has helped to increase mutation rates in rice. It demonstrates that increasing genetic variability is one target of the molecular studies, for both functional discoveries and for the manipulation of agronomically important traits. However, the question remains, have we already created enough genetic variability for the development of a super rice? Certainly, research and the development of new technologies will continue to emerge promoting the mutation induction application for superior rice genotype development.

**Figure 8:**
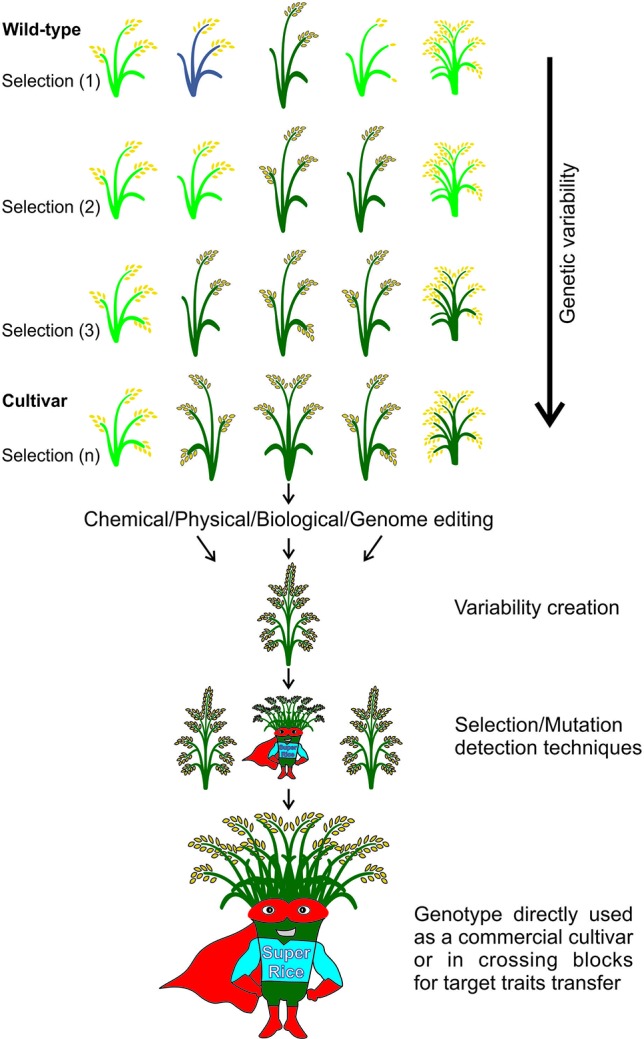
Strategies to generate variability in rice. The improved cultivars need to fulfill requirements, i.e., be adapted to the growing environments and please consumer taste. With the long-time selection for desired traits, the genetic variability is reduced, and genotypes decrease their value due to adaptation (i.e., environments change) or consumer taste (i.e., consumers change their habits) losses. To overcome this problem, the strategies of random or targeted mutations are applied to increase rice genome variations, generating new populations with different traits that can be selected according to the aim of the breeder. With this, the aim is to produce a super rice, which responds to environmental and market demands.

In any future scenario, rice breeders will always have a defined goal, increasing yield, to ensure the food security of a constantly growing population. Allied to this, there is a demand for smart crop varieties, tolerant to adverse environmental conditions in a climate changing planet. Still, there are efforts to increase rice quality, grain appearance, milling properties, eating and cooking qualities, sensory and nutritional composition, in order to ensure consumer and farmer acceptance, and supply the demand for vitamins and minerals in populations where rice is the main source of food. Based on the studies discussed here, it is possible to verify that mutagenesis is a key tool in the development of genetic resources, which are new sources of variability for the development of varieties, to overcome future demands (**Figure 8**). Thus, different agents for mutagenesis (target or random) can be considered revolutionary tools in plant breeding. Our breeding efficiency has increased due to better genomic resources and other advances in molecular genetics, cellular biology, and phenotyping techniques. Considering these aspects, mutagenesis had, has and will certainly have an impact in rice genetics and breeding.

## Author Contributions

AC developed the initial concept and outline. VE, CP, and CB improved the proposal and the manuscript. VE, CP, CB, and AC edited the manuscript. All authors read and approved the final manuscript.

## Funding

The research was supported by the Coordenação de Aperfeiçoamento de Pessoal de Nível Superior (CAPES), Fundação de Amparo à Pesquisa do Estado do Rio Grande do Sul (FAPERGS), and the Conselho Nacional de Desenvolvimento Científco e Tecnológico (CNPq).

## Conflict of Interest

The authors declare that the research was conducted in the absence of any commercial or financial relationships that could be construed as a potential conflict of interest.
